# Commentary: Malnutrition and nutritional deficits as aggravating factors in Guillain-Barré syndrome: a call for nutritional intervention in the Gaza Strip

**DOI:** 10.3389/fpubh.2025.1724066

**Published:** 2025-12-10

**Authors:** Monica Saini, Thirugnanam Umapathi

**Affiliations:** 1 National Neuroscience Institute, Singapore, Singapore; 2 Singapore National Eye Centre, Singapore, Singapore

**Keywords:** malnutrition, Guillain-Barré syndrome, beriberi, neuropathy, thiamine

We welcome the publication of this poignant paper (1). It has added to the growing literature on the importance of malnutrition in determining the outcome of patients with Guillain-Barre syndrome (GBS) in under-resourced settings (2). We had previously written on the mimicry of GBS by acute beriberi neuropathy (3). Malnourished individuals, especially if they are given a large and sudden carbohydrate load, may develop acute flaccid paralysis resembling GBS. We would like to remind your readers the need to consider this differential diagnosis and timely administer large amounts of parenteral thiamine. The key points that favor beriberi neuropathy over GBS are longer than 3 weeks progression of symptoms, alterations in mental state, nystagmus, vocal cord dysfunction, heart failure [volume overload], unexplained raised serum lactate, nil cytoalbuminergic dissociation, and the absence of the sural sparing pattern on nerve conduction studies (4). Finally, we would like to thank Dr. El Bilbeisi and his colleagues for their sterling work in the catastrophic and preventable famine conditions of Gaza.
